# Survey of Rehabilitation Clinicians in the United States: Barriers and Critical Use-Cases for mRehab Adoption

**DOI:** 10.1007/978-3-030-58805-2_30

**Published:** 2020-08-12

**Authors:** John Morris, Nicole Thompson, Tracey Wallace, Mike Jones, Frank DeRuyter

**Affiliations:** 8 Institute Integriert Studieren, JKU Linz, Linz, Austria; 9 Jack Baskin School of Engineering, UC Santa Cruz, Santa Cruz, CA USA; 10 Dipartimento di Meccanica, Politecnico di Milano, Milan, Italy; 11 Support Centre for Students with Special Needs, Masaryk University Brno, Brno, Czech Republic; 12 Crawford Research Institute, Shepherd Center, Atlanta, GA 30309 USA; 13 Duke University Medical Center, Durham, NC 27705 USA

**Keywords:** eHealth, mHealth, Clinician attitudes, Technology

## Abstract

This paper presents data and analysis from survey research conducted by the Rehabilitation Engineering Research Center on Information and Communications Technology Access for Information and Communications Technology (ICT) Access for Community Living, Health and Function (LiveWell RERC) on the perceptions and attitudes of clinical professionals in rehabilitation medicine regarding mobile health (mHealth) and mobile rehabilitation (mRehab) practices, techniques and technology in the United States. The analytical focus of this paper is on two key survey questions related to specific barriers and opportunities (most critical use-cases) for adopting mHealth/mRehab interventions. We present response data to these two questions segmented by clinical specialty – physical, occupational, speech and recreation therapy – to identify possible variation between and among these rehabilitation professions. This analysis provides a detailed map of the terrain of clinician expectations and experiences for the adoption and implementation of mHealth/mRehab interventions in the United States, and possibly other countries. Results show substantial support for mRehab interventions and technologies across all four clinical specialties. The most frequently identified barriers to effective use of mobile and internet technologies to support patients remotely focused on patients (ability to learn and use the technology, and internet access), not clinicians. The was more variability among clinical specializations regarding best use-cases. Tracking patient adherence to prescribed activities and supporting patients in the home and community were the most frequently cited best use cases across the whole sample.

## Introduction

This paper presents data and analysis from survey research conducted by the Rehabilitation Engineering Research Center on Information and Communications Technology Access for Information and Communications Technology (ICT) Access for Community Living, Health and Function (LiveWell RERC) on the perceptions and attitudes of clinical professionals in rehabilitation medicine regarding mobile health (mHealth) and mobile rehabilitation (mRehab) interventions and technology in the United States. We focus on two key survey questions related to specific barriers and best use-cases for mRehab interventions identified by clinicians:

- What barriers might limit or detract from mobile and internet technology’s effectiveness in supporting post-acute and between-visits therapy interventions?
- What do you believe are the most critical use cases for mobile or internet technology support in post-acute or between-visits therapy interventions?

We provide summary results from all respondents and analysis of responses by four core clinical specializations: physical, occupational, speech and recreation therapy to identify possible variation between clinical professions. This analysis provides a detailed map of the terrain across multiple dimensions for the adoption and implementation of mRehab interventions in the United States, and possibly other countries.

## Background

Mobile health and mobile rehabilitation (mHealth and mRehab) services and technologies have attracted considerable interest from healthcare providers, technology vendors, rehabilitation engineers, investors and policy makers in recent years [1–3]. Successful adoption and use of mHealth/mRehab interventions requires clinician support and engagement, including the ability to identify appropriate use cases and possible barriers to use for rehabilitation clinicians and their patients, and acquire adequate knowledge and confidence using mHealth/mRehab interventions. We present results from a survey of rehabilitation clinicians in the United States on their attitudes, experience, expectations, and concerns regarding mHealth/mRehab interventions and technologies. Over 500 clinicians in physical, occupational, speech, recreation therapy professions, among others, participated in the survey.

Mobile healthcare and mobile rehabilitation offer the potential to dramatically expand services to patients in need. Indeed, the World Health Organization (WHO) views “digital health” solutions as a key tool to support the goal of Universal Health Coverage (UHC). According to the WHO: “Digital technologies provide concrete opportunities to tackle health system challenges, and thereby offer the potential to enhance the coverage and quality of health practices and services” [4].

This view is reflected in the World Federation of Occupational Therapists (WFTO), whose Statement of Position states: “Telehealth is an appropriate delivery model for occupational therapy services when in-person services are not possible, practical, or optimal for delivering care and/or when service delivery via telehealth is mutually acceptable to the client and provider” [5]. In the United States the main professional organization for each of the 3 core therapy specializations (physical, occupational and speech therapy) have published statements that support use of telehealth/mHealth [6–8]. Like the WHO and WFTO, these organizations emphasize the ability to extend therapy services to more patients in a more flexible way to the home and community by using technology-supported interventions.

## Methodology

With input from our clinical advisors and informed by a review of the available literature on barriers to adoption of digital health interventions, we developed the “Clinician perspectives on mRehab interventions and technologies” survey questionnaire consisting of 22 questions to address four broad topics:

1. Perceived need for mRehab interventions based on patients’ therapy needs post-discharge from inpatient rehabilitation or between outpatient clinic visits
2. Perceived barriers to use of mRehab, including personal interest or reservations about mRehab interventions
3. Perceptions regarding the potential utility of mRehab interventions including the most important use cases for the technology
4. Current interest in, knowledge about, or actual experience using mRehab strategies

Data were collected from January 22 to March 10, 2019. Participants were recruited through the researchers’ personal networks at Shepherd Center, Duke University Medical Center, the American Congress of Rehabilitation Medicine, American Physical Therapy Association, American Occupational Therapy Association, American Speech-Hearing Association, and others. Data were collected using convenience sampling methods and online data collection on the Survey Monkey web-based platform. Although no protected health information (PHI) was collected in this survey, the Survey Monkey platform does meet the privacy and security requirements of the United States Health Insurance Portability and Accountability Act of 1996 (HIPAA), which establishes essential policies and practices for protecting patient health information from unnecessary and unauthorized access.

Efforts were made to ensure that relative balance in the number of respondents among the 4 core clinical therapy professions (physical, occupational, speech therapy and recreation therapy) by creating unique “collectors” in Survey Monkey and setting limits on the number of respondents to each. A small incentive—a $5.00 Starbucks gift card sent electronically—was offered to respondents to encourage higher levels of completeness in survey responses. The Research Review Committee at Shepherd Center reviewed and approved this research to ensure protection of participants.

## Results

Response data were analyzed using SPSS version 22. A total of 505 rehabilitation clinicians across multiple rehabilitation specialties completed the questionnaire. About half of respondents reported between 5 and 19 years of experience in their profession, and slightly more than half (55%) personally owned a wearable fitness tracker, smart watch, or other wearable device with sensors. Table 1 provides a summary of survey respondents by profession. The “Other” category includes physical therapy assistant, certified occupational therapy assistant (COTA), medical assistant, rehabilitation instructor, experimental psychologist, and others.

**Table 1.** Respondents by profession (number and percentage of sample).

Disability type	Number	Percent
Physician	13	2.6
Non-Physician Medical (Phys. Asst, Nurse Practitioner, Nurse)	13	2.6
Physical Therapist	72	14.3
Occupational Therapist	104	20.6
Speech-language Pathologist	166	32.9
Recreational Therapist	57	11.3
Mental Health (Psychologist or Counselor)	54	10.7
Other professions	26	5.1

Many respondents reported treating multiple patient populations including those with acquired brain injury (ABI), neurodegenerative diseases (NDD), musculoskeletal injury or disorder, cardiovascular disease (CVD), cancer, spinal cord injury (SCI) and other conditions (Table 2).

**Table 2.** Respondents by rehabilitation population served (number and percentage of sample).

Disability type	Number	Percent
Acquired Brain Injury (ABI)	375	74.3
Neurodegenerative Disease (NDD)	300	59.4
Musculoskeletal injury/disorder	198	39.2
Cardiovascular Disease (CVD)	181	35.8
Cancer	178	35.2
Spinal Cord Injury (SCI)	172	34.1
Other populations	119	23.6

Similarly, many respondents reported working in multiple clinical environments, including inpatient and outpatient environments, as well as skilled nursing facilities, home health and other environments (Table 3).

**Table 3.** Clinical environments of respondents (number and percentage of sample).

Disability type	Number	Percent
Inpatient acute	146	28.9
Inpatient rehab	203	40.2
Outpatient clinic	243	48.0
Skilled nursing facility	72	14.3
Home health	48	9.5
Other environments	73	14.5

To map the challenges and opportunities for implementing new mHealth and mRehab interventions for people with disabilities and chronic conditions, respondents were asked to: 1) select the 3 most likely barriers or concerns that might limit or detract from the effectiveness of mobile and internet technology to support post-acute and between-visits therapy interventions; and 2) to identify the 3 most critical use cases for technology-based remote interventions.

For each question, respondents were provided a list of barriers and use cases, respectively, with the option to specify additional barriers and use cases in an open-ended comment field. Tables 4 and 5 show the response rates for barriers and use cases for the core therapy specializations (physical, occupational, speech), plus the small but growing field of recreation therapy.

**Table 4.** What barriers might limit or detract from mobile and internet technology’s effectiveness in supporting post-acute and between-visits therapy interventions? (Select 3).

Barriers to use	Physical therapy	Occupational therapy	Speech therapy	Recreation therapy	All 4 specializations
Patients unable to learn and/or correctly use technology	72.2%	77.9%	78.3%	73.7%	76.4%
Patients with limited or no access to internet services	50.0%	64.4%	74.1%	63.2%	65.7%
Cost vs. reimbursement	26.4%	36.5%	35.5%	22.8%	32.8%
Hassle and time commitment for clinicians to adopt	36.1%	26.9%	18.7%	19.3%	24.1%
Patient concern over security and privacy	22.2%	19.2%	23.5%	33.3%	23.6%
Concerns over accuracy and reliability	31.9%	18.3%	21.1%	15.8%	21.6%
Improvement in outcomes or efficiency not sufficient	12.5%	11.5%	11.4%	10.5%	11.5%

**Table 5.** What do you believe are the most critical use cases for mobile or internet technology support in post-acute or between-visits therapy interventions? (Select 3).

Use cases	Physical therapy	Occupational therapy	Speech therapy	Recreation therapy	All 4 specializations
Support patient adherence to prescribed exercises/activities	73.6%	61.5%	72.9%	57.9%	67.9%
Support patient functioning at home and in the community	51.4%	70.2%	78.9%	59.6%	68.9%
Real-time, direct observation, communication with patients	56.9%	51.9%	46.4%	50.9%	50.4%
Remote biometric monitoring of patient activity with apps or wearable tech	48.6%	33.7%	24.7%	24.6%	31.3%
Patients’ self-reporting of outcomes	33.3%	26.0%	30.7%	28.1%	29.6%
Remote environmental monitoring using sensors in homes	23.6%	27.9%	13.9%	12.3%	19.0%

Respondents in all four professions identified concerns over the ability of patients to learn and use the mRehab technology correctly (76.4% of all respondents in these professions) and access to internet services (65.7%) as the top 2 potential barriers to the effectiveness of mobile and internet technology to support their patients. Physical therapists were notably less concerned with internet access compared to the other four professions.

There was some variation in the secondary barriers identified among the four professions. Physical therapists were more concerned with the hassle and time commitment required of clinicians for set-up (36.1%), and the accuracy and reliability of the technology (31.9%). Occupational and speech therapists were more concerned with the cost of the solutions and reimbursement by insurance providers (36.5% and 35.5%, respectively). Recreation therapists identified security and privacy of the technology as their leading secondary concern (33.3%).

There was greater variation in the most critical use cases identified by respondents in each of the four therapy professions. Almost three-fourths (73.9%) of physical therapists identified supporting patient adherence to prescribed exercises and activities. Notably, a similar percentage of speech therapists (72.9%) also identified this as a critical use case. But, speech therapists’ most frequently cited use case was supporting patient functioning at home and in the community (78.9%). This was also the most frequently cited use cases for occupational and recreation therapists (70.2% and 59.6%, respectively).

Approximately half of the respondents in each profession cited real-time, direct observation and communication with patients as a critical use-case, making this the third most cited use-case. Also noteworthy, almost half of the physical therapists (48.6%) cited remote biometric monitoring as a critical use case. Patient self-reporting of outcomes and remote environmental monitoring using sensors in the home were least frequently cited overall.

## Conclusion

Clinical professionals often serve as “gatekeepers” for new healthcare technologies [9]. They are the ones who must prescribe a technology-based intervention to a specific patient, or not. Within a large clinical facility (hospital or large clinic), clinicians are often engaged in technology reviews and planning for implementation of new patient-care technologies. Consequently, it is critical to identify their perceptions of the barriers and best uses for new technologies such as those used for mRehab.

The survey data presented here on the perceptions of rehabilitation clinicians indicates broad acceptance of mHealth technologies and interventions for specific use-cases. There was consensus among respondents in the four rehabilitation therapy professions analyzed here in favor of using of mHealth technologies to support patient adherence to prescribed exercises and activities and to support patient functioning in the home and community. There was much less support for use-cases involving wearable or environmental sensors and patient self-reporting.

There was strong consensus that the main barriers to adoption related to *the patients*, not the clinicians. Patients’ ability to learn and use the technology correctly and patient access to the internet were the top perceived barriers. Other potential barriers such as reimbursement from insurance providers, hassle and time commitment on the part of clinicians, security and privacy, accuracy and reliability of data collected, and improvement in health outcomes were much less frequently identified as barriers.

The low levels of concern for these other potential barriers is noteworthy, as these have been identified as concerns in earlier studies of physician perspectives on digital health. In a 2016 survey of physicians in the U.S., most respondents reported being concerned about potential liability, reimbursement, technical problems, and patient privacy [10]. Similarly, a survey of physicians in Europe cited patient privacy and data security as major concerns [11]. A scoping review of the literature on physician attitudes toward eHealth conducted by Canadian researchers identified technology design, training, liability, and patient privacy as key issues [12].

It is possible that rehabilitation therapists are more likely to utilize a wider range of technologies, especially mobile and internet technologies, than physicians. Also, rehabilitation therapy usually involves more frequent and longer-duration interactions with patients. That degree of engagement with patients may motivate rehabilitation clinicians to be more concerned about patient access than they might be regarding reimbursement, time commitment on the part of the clinician, privacy, etc.

The survey of clinician perspectives on mRehab interventions and technologies serves as the cornerstone for our long-term commitment to engage clinicians regularly regarding their experiences using new methods and modalities of interacting with their patients and using newly emerging technologies. We plan to update and refine this cornerstone survey with the goal of tracking clinician perceptions and experiences over time. Additionally, we plan to conduct more targeted research on topics such as experiences implementing a cloud-based digital therapeutics system for prescribing home exercise programs (HEPs) and tracking progress; and using specific consumer platforms to track patient activity such as smartwatches, fitness trackers, camera-based systems, smart speakers and other smart home devices.
